# A Mixed Methods Evaluation of a Randomized Control Trial to Evaluate the Effectiveness of the Pure Prairie Living Program in Type 2 Diabetes Participants

**DOI:** 10.3390/healthcare8020153

**Published:** 2020-06-03

**Authors:** M. Carolina Archundia-Herrera, Fatheema B. Subhan, Cathy Sakowsky, Karen Watkins, Catherine B. Chan

**Affiliations:** 1 Department of Agricultural, Food and Nutritional Science, University of Alberta, 6-002 Li Ka Shing Centre for Health Innovation Research, Edmonton, AB T6G 2E1, Canada; archundi@ualberta.ca; 2 School of Public Health, University of Alberta, Edmonton, AB T6G 2T4, Canada; fatheema@ualberta.ca; 3 Sherwood Park Primary Care Network, 150 Broadway Crescent, Suite 108, Sherwood Park, AB T8H 0V3, Canada; CathyS@sherwoodparkpcn.com (C.S.); karenw@sherwoodparkpcn.com (K.W.); 4 Department of Physiology, 6-002 Li Ka Shing Centre for Health Innovation Research, University of Alberta, Edmonton, AB T6G 2E1, Canada

**Keywords:** type 2 diabetes, lifestyle interventions, Pure Prairie Living Program, nutrition

## Abstract

The primary objective of this randomized control trial was to evaluate the effectiveness of the Pure Prairie Living Program (PPLP) in a primary care setting. Adults with type 2 diabetes were randomized into intervention (PPLP, *n* = 25) and wait-listed controls (CON, *n* = 24). The PPLP group participated in education sessions. The intervention yielded no significant within-group changes in HbA1c at three-month (−0.04 (−0.27 to 0.17) and −0.15 (−0.38 to 0.08)) or six-month (−0.09 (−0.41 to 0.22) and 0.06 (−0.26 to 0.38)) follow ups in either CON or PPLP groups, respectively. Dietary adherence scores improved in the PPLP group (*p* < 0.05) at three and six months but were not different in the between-group comparison. No changes in diabetes self-efficacy scores were detected. In the qualitative analysis, participants described the program as clear and easy to understand. Knowledge acquired influenced their everyday decision making but participants faced barriers that prevented them from fully applying what they learned. Healthcare professionals enjoyed delivering the program but described the “back-stage” workload as detrimental. In conclusion, while some positive effects of the PPLP intervention were observed, they were not comparable to those previously attained by our group in an academic setting or to what the guidelines recommend, which reflects the challenge of translating lifestyle intervention to real-world settings.

## 1. Introduction

Obesity has been present since stone-age times and throughout the major eras of history [1]. Its social context has evolved from being associated with well-being and power to now being a health problem and an epidemic that threatens global welfare [2]. Since the beginning of the 21st century, obesity has been regarded as a medical problem [3] because it is a significant risk factor for a number of cardiometabolic diseases (CMD) due to its link to chronic inflammation; disturbed cellular metabolism; increased insulin resistance; and overall metabolic dysfunction prompting metabolic syndrome (MetS), type 2 diabetes (T2D), hepatic steatosis, and cardiovascular disease (CVD) [4].

Alarming predictions suggest that in the USA, the national prevalence of adults with obesity (BMI 30 to 35) and severe obesity (BMI > 35), by 2030, will be three in four adults. Therefore, combined obesity and severe obesity should be the most common BMI category nationwide among women, black non-Hispanic, and low-income adults [5]. In a similar manner, globally, the prevalence of T2D is predicted to increase from 8.8% in 2015 to 10% in 2030, accompanied by a corresponding 61% increase in economic burden to USD $2.1 trillion for diabetes and its complications [6]. Amelioration of the problem could be achieved by tackling suboptimal diet, which contributes an estimated annual CMD cost, in the USA, of USD $50.4 billion [7]. Although a wide variety of approaches to cure, treat, and prevent CMD are being investigated globally, a general consensus to prioritize the prevention and reduction of modifiable risk factors is difficult given the focus on associated comorbidities, which are major health and financial problems [5,6]. 

Clinical practice guidelines have been developed to provide evidence-based care for people with CMD, in particular in the fields of CVD and T2D through specific diet, physical activity, and pharmaceutical recommendations [8,9,10,11]. However, people tend not to meet dietary and physical activity recommendations, the main barrier being the complexity of translating the guidelines into actions, and in general, understanding what they mean [12]. Gaps in knowing how to address or overcome obstacles to behavior change include “family influence, perception of healthy food being not tasty, lack of skills to prepare or choose healthy food, difficulty in finding healthier options when eating out, and healthy food being costly” [13]. 

Previously, our group has worked on bridging the guideline-practice gap through the development of a four-week menu plan focused on general dietary habits of people living in Alberta, Canada, translating the Diabetes Canada (DC) Nutrition Therapy Guidelines [14] into a concrete menu plan and a recipe book based on the 4A framework, i.e., food availability, accessibility, acceptability, and adequacy [15]. A pilot study was conducted to evaluate the feasibility and efficacy of menu planning combined with individual counseling on healthy eating for improving health outcomes and it yielded improved HbA1c and dietary adherence score [16], encouraging a larger trial. The Physical Activity and Nutrition for Diabetes in Alberta (PANDA) single-arm trial focused on group education sessions to help people incorporate DC Nutrition Therapy Guidelines into their daily lives through the use of menu planning and an educational program, with the primary objective of evaluating the effectiveness of the intervention on HbA1c and dietary adherence among patients with T2D [17]. Both studies demonstrated that the menu plan and recipe book could be a simple and practical resource, combined with education and behavior change strategies, for improving adherence to nutritional recommendation guidelines [16,17].

The end goal of research findings is not complete until the knowledge acquired in research is fully applied in real-world settings [18]. Thus, we aimed to test our PANDA program in a primary health care setting to validate our previous results and further refine the programming to achieve real societal impact. Hence, the primary objective of this study was to evaluate the effectiveness of the Pure Prairie Living Program (PPLP), a lifestyle intervention tailored to Albertans, in improving diabetes management (HbA1c) and promoting better nutrition choices (dietary adherence to the guidelines, diabetes self-efficacy) in a primary care network (PCN) setting. To evaluate thoroughly the PPLP intervention beyond clinical parameters, a mixed-methods approach was undertaken to understand the perceived effectiveness and limitations of the PPLP contextualized by health care providers (HCP) and study participants. 

## 2. Materials and Methods 

### 2.1. Study Design

The PPLP intervention (ClinicalTrials.gov ID: NCT03043859) was a 2-arm, parallel group, randomized controlled trial. Participants were blinded to group assignment through concealment of allocation until assignment occurred using a simple random table. Participants were randomized 1:1 to one of the groups (intervention (PPLP) or control (CON)). This educational program was originally designed to be implemented at 2 PCNs in Edmonton, AB, Canada, with the aim of recruiting 120 participants with 60 participants from each site (total 60 participants in each arm); however, after the implementation of the program at the first PCN, the study team came to a consensus to modify the original protocol to work with 1 PCN (total 60 participants, 30 per arm). The rationale behind this decision lies in the fact that the PPLP is part of a multistage study design that uses accumulating data. Thus, input from the first center directs modification of aspects of the PPLP. 

### 2.2. Participants: Eligibility and Recruitment

Eligible participants in the PPLP were adults (30–80 years) with T2D (self-identified) able to read and write in English, willing to commit to the study, and able to attend weekly meetings. Exclusion criteria were participants not able to read and write in English, pregnant or breastfeeding women, participants with type 1 diabetes or with medical comorbidities or severe diabetes complications requiring a highly specialized diet or living in long term care (LTC), and unable to provide consent (e.g., cognitive impairment). Two recruitment methods were followed. For the first method, a convenience sampling procedure was employed to recruit study participants through advertisement and posters at the PCN. Interested individuals contacted the study coordinator via phone and were recruited to the study if they met the inclusion criteria described above. For the second method, the electronic medical record (EMR) was used to short-list participants with T2D in the target age range (*n* = 338) and their charts were screened for eligibility. Those who fulfilled the inclusion criteria were contacted via phone call. Those interested were recruited to the study.

### 2.3. Study Setting

The study took place at Sherwood Park PCN, Edmonton, AB, from May to November 2017 (PPLP group) and January to February 2018 (CON). In Alberta, PCNs provide citizens with access to a multidisciplinary care team of clinicians, including dietitians. This study was conducted according to Canadian and International Standards of Good Clinical Practice for all studies. The University of Alberta Research Ethics Board approved the protocol for this study (Study ID Pro00070736). 

### 2.4. Study Intervention

**Figure 1:**
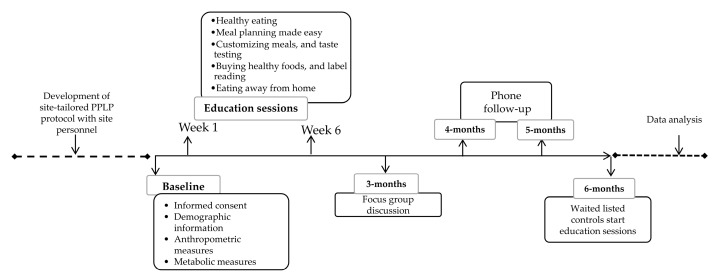
Intervention timeline.

The primary goal of this study was to evaluate the efficacy of the PPLP in improving glycemic control (HbA1c) and other risk factors for T2D. The intervention group participated in the PPLP group educational sessions. These sessions were conducted at the Sherwood Park PCN facilities and facilitated by two of the PCN registered dietitians (RDs). Site personnel were co-investigators in the research to facilitate maximal buy-in and integrated knowledge translation (KT). Prior to implementation of the study, the research personnel provided on-site training in the PPLP to the RD, reviewed materials and resources, and worked in conjunction with the PCN team to develop a site tailored PPLP intervention protocol (Figure 1).

Modifications to the educational materials were made with consensus between researchers and practitioners in order to make some elements consistent with other programming offered by the PCN while maintaining the foci of the original PANDA trial [17]. The facilitators were provided with lesson plans for each education session to ensure standardized delivery of the education session across facilitators and to increase delivery fidelity. The intervention curriculum follows best practices in nutrition interventions for patients with diabetes using the social cognitive theory as the overall theoretical model to guide behavioral change [17]. 

#### 2.4.1. Intervention Study Arm

Participants in the intervention study arm attended and participated in group education sessions (~90 min each) during a six-week period. They received the PPLP resource package that consisted of the following:

**Education sessions:** Participants received a copy of the nutrition education presentations each week. The PPLP curriculum is based on the PANDA program which has been described elsewhere [17]. Briefly, the presentations included information to enhance knowledge of healthy eating based on practical information derived from Eating Well with Canada’s Food Guide [19] and Diabetes Canada Clinical Practice Guidelines for Nutrition Therapy [14]. Participants learned to apply principles of menu planning, grocery shopping, portion control, label reading, and making healthy choices when eating out. A detailed summary of intervention activity content, presentations of group-based educational sessions, and support materials can be found at www.pureprairie.ca/resources [20].
**PPLP workbook:** Participants were provided with a workbook that guided them through the educational sessions. It provided support, positive reinforcement of concepts, and the opportunity for skill acquisition through the practice of goal setting, self-monitoring, and problem solving. This aligned with the principles of the social cognitive theory theoretical model.
**Pure Prairie Eating Plan:** Participants were provided with a copy of the Pure Prairie Eating Plan (PPEP). This four-week menu plan book meets the DC nutrition therapy guidelines [14] and is guided by the principles of the 4-A framework. Its overall goal is to facilitate solutions to some of the barriers that participants face when trying to adhere to the recommendations. Additional information on the PPEP is available at www.pureprairie.ca [21].

#### 2.4.2. Wait-List Control Group 

Participants in this group were required to attend three assessment sessions (baseline, 3-month, 6-month) after which they were offered the PPLP educational sessions delivered by the same RDs as for the PPLP group. No further assessments were done. This procedure was chosen in order to guarantee equitable treatment for all participants enrolled in the study, thus all participants had access to the program.

#### 2.4.3. Study Outcomes 

The primary outcome for this study was change in HbA1c. The secondary outcome was changes in the Perceived Dietary Adherence Questionnaire (PDAQ) and Diabetes Self Efficacy Scale (DSES) scores. Assessments were performed at three different time points (baseline, 3-month, 6-month) at the Sherwood Park PCN (Figure 1). All participants received a phone call to remind them of their upcoming appointment. The same instruments were used for all the measurements throughout the study.

#### 2.4.4. Anthropometric Measures 

Body weight and height were measured to the nearest 0.1 kg and 0.1 cm, respectively, to calculate body mass index (BMI). Waist circumference was measured to the neared 0.1 cm with the participant in a standing position, with a non-stretch tape placed midway between the lateral lower ribs and the iliac crest after a moderate expiration. Body composition was determined using a BIA scale (Tanita, Arlington Heights, IL, USA). Differences in weight, BMI and waist circumference (baseline to 3 months, baseline to 6 months, and 3 months to 6 months) were calculated.

#### 2.4.5. Metabolic Measures

Blood pressure was assessed with participant seated using an auto-inflated digital unit (BpTRU, Model BPM-100, Coquitlam, BC, Canada). Non-fasted finger prick blood samples were collected. Briefly, with the participant’s hand and arm in a horizontal position, the middle or ring finger of the non-dominant hand was cleaned with an alcohol wipe. The end of a lancet was placed against the fingerstick site (top or bottom) and pushed firmly, then pressure was applied gently on the finger to collect two samples of blood. The point-of-care testing devices used 5 μL of blood for HbA1c (Bayer, Elkhart, IN, USA) and 20 μL for lipid profile (triglycerides (TG), total-cholesterol (TC), low-density lipoprotein cholesterol (LDL-C) and high-density lipoprotein cholesterol (HDL-C)) (CardioCheck, Whitestown, IN, USA). Blood was collected using PTS capillary tubes. The blood samples were applied to the analyzers within 30 s of collection. Differences in test outcomes pre- and post-intervention were calculated.

#### 2.4.6. Demographic Characteristics and Additional Self-Reported Data

All participants (CON and PPLP group) provided sociodemographic data at baseline and completed the following validated questionnaires at baseline and each follow up. The Perceived Dietary Adherence Questionnaire (PDAQ) (10 items) measured self-reported adherence to dietary recommendations by DC Nutrition Therapy Guidelines and Eating Well with Canada’s Food Guide. Each item was scored 0–7 (0 = poor adherence and 7 = maximum adherence) with items 4, 9, and 10 scored using “reverse scoring”. These results were averaged yielding a score of 0–7. The Diabetes Self-Efficacy Score (DSES) (8 items) was used to measure self-reported changes in nutrition self-efficacy. Each item was scored 1–10 (1 = not confident and 10 = totally confident), then divided by 8, yielding a score of 0–8.3. Quality of Life (EQ-VAS) recorded the patient’s self-rated health on a vertical visual analogue scale corresponding to a score 0–100 (0 = the worst health you can imagine and 100 = the best health you can imagine) reflecting participants’ own judgment.

#### 2.4.7. Qualitative Assessment 

During the three-month visit, two focus group discussions were conducted with PPLP participants and two semi-structured, individual interviews with HCP. The focus groups and interviews were conducted at Sherwood Park PCN and were 30 to 45 min long. The overall aim was to understand participants’ experiences throughout the program including knowledge about managing diabetes, barriers perceived, and suggestions with the aim of improving the overall program using a thematic analysis approach. Transcripts of focus groups and interviews were transcribed verbatim and analyzed using Nvivo software version 11.4. The transcripts were checked against the recordings for accuracy. When conducting the thematic analysis, Brauns’ suggested step-by-step guide [22] was used to ensure rigor. Morse’s verification strategies were also acknowledged [23]. Briefly, first, familiarization took place by reading the data in an active way and taking note of initial ideas. Second, the entire set of data was organized into meaningful groups and initial codes were created. In the third and later stages of coding, the initial codes were classified into potential themes using an inductive approach followed by reviewing the initial themes to make sure they accurately reflected the whole dataset. The last phase involved defining and naming themes for the final analysis. 

#### 2.4.8. Sample Size

Previous literature has indicated that nutrition therapy and lifestyle modifications could improve hyperglycemia in T2D patients by 1%–2% [14]. Our research team’s previous single-arm phase 2 trial with 73 participants with T2D demonstrated a 0.7% decrease in HbA1c (95% CI, 0.4% to 1.0%, *p* < 0.05) at three months after the intervention [17]. Because the primary objective of the present study was to evaluate the effectiveness of PPLP on HbA1c levels, the sample size was calculated with the aim of detecting a 0.5% change in HbA1c, which is clinically meaningful. Statistical power was considered as 0.80 and alpha = 0.05, thus sample size was estimated as 25 in each group with an estimated dropout rate of 20%. 

#### 2.4.9. Statistical Method

The Statistical Package for the Social Sciences (SPSS) software version 24 (SPSS Inc., Chicago, IL, USA) was used for the following analyses: Kolmogorov–Smirnov and Shapiro–Wilk tests were used to determine distribution of variables; between groups, for continuous variables, independent t-test comparison of baseline, 3- and 6-month characteristics (demographic, anthropometric and metabolic) or, for categorical variables, Chi-square test; and paired t-test was conducted to analyze the changes over time within groups. Results are presented as mean group differences with 95% confidence intervals (CI). Statistically significance was set for two-tailed *p* values of <0.05. Significant differences in parameters are reported as * *p* < 0.05 and ** *p* < 0.001. 

## 3. Results

**Figure 2:**
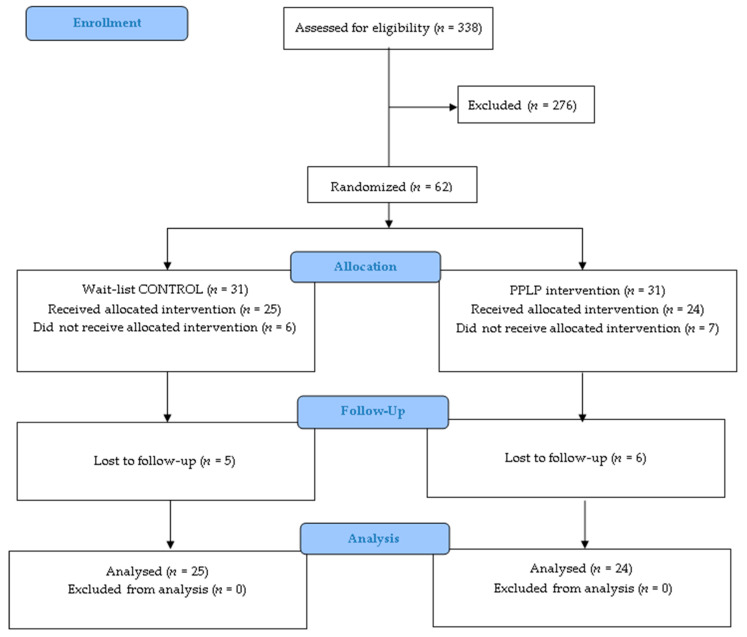
CONSORT flow diagram.

All 62 respondents met the inclusion criteria and were randomized to either the wait-listed control (CON *n* = 31) or the intervention groups (PPLP *n* = 31) (Figure 2). Forty-nine participants attended the baseline meeting, provided signed consent, and received the allocated intervention; 42 participants were assessed during the 3-month follow up; and 39 participants completed the 6-month follow up. 

### 3.1. Quantitative Outcomes

#### 3.1.1. Baseline Characteristics

The demographics characteristics of the study participants are presented in Table 1. At baseline, there were no between-group differences in gender, age, or duration of diabetes. The majority of the participants (87.8%) used antidiabetic drugs, and/or insulin combined with diet and/or exercise to manage their diabetes. Metformin was the medication most commonly used by the participants (76.7%). In addition to T2D, participants had a mean of two concurrent illnesses with the highest prevalence being hypertension. Likewise, 79.1% and 60.5% reported taking medications to treat hypertension and hyperlipidemia, respectively. Baseline anthropometric and metabolic parameters of participants were analyzed separately by gender. As shown in Table 2, there were no statistically significant differences between PPLP and CON with the exception of systolic blood pressure (SBP) in men (Table 2).

#### 3.1.2. Changes in HbA1c (Primary Outcome)

There were no significant within-group changes in HbA1c or lipids observed at either 3- or 6-month follow ups, nor were between-group differences detected (Table 3).

#### 3.1.3. Intervention Effects on Diet Adherence and Self-Efficacy (Secondary Outcomes) 

The PDAQ and DSES scores for both CON and PPLP groups are presented in Table 4. At 3- and 6-month assessments, the PPLP participants improved their PDAQ (*p* < 0.05). These improvements were not different in the between-group comparison. No change in DSES score was detected (Table 4).

#### 3.1.4. Intervention Effects on Anthropometric and Metabolic Parameters (Exploratory Outcomes)

The analysis of mean change within groups from baseline to 3- and 6-month follow ups revealed a decline in BP for both groups (Table 3) that was not different between groups. In the PPLP group, body weight decreased significantly at both 3- (** *p* < 0.001) and 6-months follow ups (* *p* < 0.05), with the between-group difference trending to benefit the PPLP group at 6-months (*p* = 0.069). Conversely, the CON group experienced a decrease in waist circumference at 3-months that was not sustained at 6 months. Furthermore, a significant decrease in fat free mass (kg) was observed in the PPLP group at 3- and 6-month follow ups (* *p* < 0.05). This effect was further observed in the 6-month between group analyses (Table 3).

#### 3.1.5. Intervention Effects on Quality of Life (Exploratory Outcomes)

Participants in both CON and PPLP groups showed an improvement in EQ-VAS score at three months. This improvement was maintained at six months by the CON group (* *p* < 0.05). 

### 3.2. Qualitative Outcomes

Two focus groups (*n* = 17) with participants and two one-on-one interviews (*n* = 2) with HCP were conducted to understand their experience throughout the program. For the focus groups, results were grouped into facilitators/barriers and experience evaluation. Facilitators are factors perceived by participants that influenced their decision-making process for applying what was learned throughout the program in their everyday lives. Barriers are factors preventing participants from applying what was learned in the program to their daily lives. Experience evaluation reports participants’ satisfaction, limitations, and improvements (denoting tangible program modifications participants would like) after participating in the PPLP. Themes and representative quotes for these categories are presented in Table 5 and Table 6.

#### 3.2.1. Facilitators and Barriers

##### Facilitators

Two themes reflected what facilitated participants’ decision making about applying what was learned throughout the program: knowledge acquired and motivation. Knowledge acquired throughout the program, in particular healthy eating, label reading, and physical activity influenced participants’ everyday life. Furthermore, health, family, and group support were the main motivators for participating in the program. 

##### Barriers 

The presence of complications that participants discussed were aligned with and supported what has previously been described as the “4 Ms framework” [24]. Mental, mechanical, metabolic, and monetary (including time) categories identify the issues participants faced throughout the program and in their everyday life (Table 5).

#### 3.2.2. Experience Evaluation

In order to improve the program, we asked participants what they liked, did not like, or how and what would they add/change to the program, with the aim of obtaining a better understanding of their needs and expectations.

##### Satisfaction

In general, participants reported an overall positive experience. They emphasized that the program was clear and easy to understand and, most importantly, realistic. 

##### Limitations 

A recurrent request was to modify or eliminate the workbook. Improvements: Potential improvements in areas including longer support from peer groups, more hands-on activities (cooking lessons), and active physical activity programs (exercise groups) were recommended (Table 6).

#### 3.2.3. HCP Perspective

Since these types of interventions were normally carried out by front-line HCP, obtaining their perspective on the overall program, logistics, delivery, satisfaction, and barriers was essential. Among the themes that could be distinguished, satisfaction, strengths, and effectiveness stood out and are presented in Table 7. The usefulness of the PPEP for helping to guide the overall intervention was emphasized as a strength of the program. The HCP stated that having the support of another peer and the research group was essential. Nonetheless, although the delivery of the program was described as enjoyable, the workload regarding the “back-stage” preparation was sensed as detrimental because it took a lot of their time, especially for participant recruitment, phone call follow ups, reminders and general administrative coordination. Furthermore, HCPs thought that the workbook needed to be simplified/modified so that participants would use it and benefit from it as a teaching tool. The importance of participant’s active role was noted as crucial for them to get the most out of this or any program.

## 4. Discussion

Lifestyle interventions evaluated in research settings have frequently been shown to improve diabetes- and hepatic steatosis-related outcomes [25,26,27,28], and are effective for the prevention and treatment of obesity and T2D complications [29]. They have a positive effect on health and are capable of reducing the incidence of T2D among people with pre-diabetes [30] in a cost-effective manner, because delaying the onset of T2D for 10 years with lifestyle interventions could save USD $30,000 in lifetime medical spending per person [31]. In Alberta, the PCNs’ main goal is to increase the emphasis on health promotion, disease and injury prevention, and care of patients with complex problems or chronic diseases. Therefore, the PCN setting was ideal for implementation of the PPLP intervention because it provided a representative real-world setting. 

Our data indicate that the PPLP offered in a real-world PCN as compared with usual care during a six-month follow up was not statistically significant or clinically relevant in relation to our primary outcome, HbA1c. A major factor, as reflected by the demographic characteristics and baseline HbA1c of the participants, was that our “real-life” participants were mostly middle class highly-motivated Caucasians who entered the study with overall good control of blood sugar (HbA1c = 6.9%) [32]. Previous evidence corroborates that patients who enrol in PCN-delivered interventions tend to be receptive, motivated [33], and better managed [34]. Our sample size calculation was based on the primary aim to detect a 0.5% change in HbA1c, however a 0.5% change in the difference between 11 and 10.5 is not the same as 8 and 7.5. In other words, achieving changes in glycemic control in patients with poorly controlled hyperglycemia can be feasible with intensive therapy and lifestyle changes versus improving glycemic control in patients with well managed tightly regulated glycemic control can be difficult to achieve and can include potential risk for hypoglycemia [35]. Our results reflect, in part, what is known as “a ceiling effect”, which occurs when baseline performance is nearly as good as it could be in both the treatment and control conditions. Furthermore, accordingly to systematic reviews, benefits are more pronounced in individuals with poor blood glucose control [33,36].

Regarding our secondary and exploratory outcomes, some improvements were observed, but not to the extent as observed in a study of T2D participants (*n* = 73) previously conducted by our team in a research setting [17]. This more modest efficacy has previously been reported as a limitation of lifestyle intervention program translation [37,38]. Indeed, weight loss in the present intervention group was 1.56 kg (*p* < 0.001) and 2.43 kg (*p* < 0.05) at three and six months. Benefits of weight control through caloric restriction [14] and intermittent fasting [39] have been reported to improve glucose metabolism through reduction of BMI, decreased circulating leptin, and increased adiponectin [39]. 

Qualitative analysis is an important part of behavioral intervention research [40]. Our findings contribute to public health prevention and management of CMD by providing essential participant perspectives. Thematic analysis was used to reflect reality [22]; it served our objective to understand participants’ experiences throughout the program including what worked, what did not work for them, and how to improve the overall program. In general, participants reported enjoying taking part of the study, it helped them increase the amount of fruit and vegetables they consumed, changed the way they perceived and acknowledged the importance of menu planning, portion control, and the consciousness about what they bought and ate. Furthermore, the qualitative analysis provided useful insight to participants’ barriers, which were consistent with previous frameworks [41], echoing the vast evidence supporting the complexity of chronic diseases [42]. Participants’ suggested improvements for future editions of the program included more hands-on activities such as cooking lessons and active exercise programs. 

Results from the PPLP intervention provide important contributions. First, HCP and PCNs are effective in the overall management of T2D as evidenced by participants’ HbA1c at baseline. Our data is corroborated by a cohort study showing that being enrolled in a PCN was associated with a 19.4% relative reduction in admission to hospital or emergency departments, potentially as a result of receiving more guideline-recommended care as compared with individuals not attending a PCN [34]. Participants who attend PCNs are prone to be more receptive and highly motivated, thereby maximizing the potential benefits they can obtain from this type of intervention [33]. Thus, participants from, underrepresented and disadvantaged groups, and those who have just been diagnosed or have poor control of their diabetes need to be identified and encouraged to enrol in these types of programs [43]. This could help to refocus and prioritize resources to target patients with poor glycemic control [44], who would greatly benefit from such programs [45]. Further research is needed to understand how to reach those most in need of such interventions, including those with a higher risk of developing T2D or T2D complications [5].

These results contribute to and further support what has previously been reported in the literature regarding outcomes of lifestyle interventions delivered in real-world settings. Transition from research into real-world settings can be less successful for a variety of reasons, particularly with regard to having less impact as compared with their research setting counterparts [46,47], and increased burden for HCP to deliver [38], raising the question of how much of an effect a lifestyle intervention must have in a research setting in order to have a clinically relevant effect in a real-world setting.

This PPLP study had some limitations. First, statistical power for secondary outcomes was limited. Post-hoc power analysis indicated that with the exception of SBP (power = 90%) this study was low to moderately underpowered to estimate differences between the PPLP and the control group. Power to estimate weight, BMI was <10% while dietary adherence, diabetes self-efficacy, and quality of life had power ≤ 50% and DBP was 70%. Using the effect sizes and variances observed in the present study can help researchers plan adequately powered studies in the future to unravel the associations between the effects of lifestyle interventions on biomedical and behavior outcomes. Furthermore, our sample was quite homogeneous; therefore, it was not representative of the general population and cannot be generalizable, although we predict an enhanced response if individuals with poorer glycemic control were enrolled. The relatively short-term follow up (six months) limited our ability to report longer-term benefits. Thirdly, some methodological limitations merit mention. Although measures were taken to reduce observer bias (training to make assessment consistent, reduce conscious or unconscious prejudices) allocation concealment was not possible. In addition, given that the questionnaires used called for participants to accurately remember previous events or experiences, risk of recall bias cannot be discarded. Eligibility and recruitment log regarding screening, eligibility, and reasons for ineligibility or not enrolling were not conducted, which was a methodological limitation of the current study but did help to reduce the workload of the HCP involved with the program and was consistent with real-world practice.

This PPLP study has several strengths. By incorporating a variety of quality improvement strategies including working with site personnel to tailor the PPLP to their setting, providing resources relevant to the Albertan population, and a detailed curriculum guide to the healthcare provider team to enhance program delivery fidelity [36], we attempted to overcome the identified limitation of general lack of replication of evidence from research to real-world settings [37]. Secondly, the use of tools specifically developed for the needs of the local community is a novel approach aimed at contributing to the prevention and management of diabetes and adoption of a healthier lifestyle. The DC Nutrition Therapy guidelines recommend the adoption of dietary patterns that have evidence for improving diabetes outcomes. One of the dietary patterns recommended is the Mediterranean dietary pattern, which is known to have multiple health benefits [14]. However, in places such as Alberta in Canada, following this dietary pattern is not always feasible given the extreme weather conditions that limit crop production, local food preferences, and other factors. Thus, the PPEP was developed to align with local reality through the use of the 4A framework, and therefore provided a dietary pattern that was acceptable to and nutritionally adequate for people in Alberta. The use of locally available and accessible food encourages the use of short supply chains, which have been linked to improved metabolic health [48]. Given the positive feedback received from participants and HCP in regard to the usefulness of the PPEP, the development of specific tools that facilitate adherence to the guidelines could be a novel approach to facilitate adherence to the guidelines and the long-term diabetes management. These tools can also reduce the workload of HCP, for example, by providing adaptable menu plans. Given that individuals are prone to engage in multiple unhealthy lifestyle behaviors [49], future interventions would benefit from using the same approach proposed in the present intervention and design tools to address other unhealthy lifestyle behaviors specific to individual participants’ context. Lastly, weight loss was not a main outcome of the PPLP trial but it was considered to be a strength of the study. Given the multifactorial nature (sedentary behavior, physical activity, sleep pattern, and stress levels) and complexity of weight management, we consider that weight loss does not necessary reflect improvement in pathophysiological mechanisms or metabolism. Rather, a focus on healthy eating can elicit more sustainable behavior change [50,51]. In order to provide new insights into the effect of lifestyle interventions on pathophysiological mechanisms in real-word settings, measuring surrogate markers of hepatic steatosis [28] or adipose-derived secretory or inflammatory factors [51] could provide more comprehensive insights.

## 5. Conclusions

In conclusion, the modest positive effects of the PPLP intervention reflect the challenge of translating lifestyle interventions to real-world primary care settings. The development of specific tools was a novel approach to facilitate and improve participants’ adherence to the guidelines and reduce HCP workload. Our results raised the important issue that the information provided in lifestyle interventions was absorbed and judged to be useful, but by itself was not enough support for participants to modify and sustain the behaviors and abilities for self-care. The PPLP allowed us to begin to understand some of the needs of participants in a general real-world setting but future research should address this gap in knowledge and investigate knowledge-to-action techniques that could improve diabetes management and ease the health journey of persons with diabetes. Further research is needed to conduct in-depth conversations with knowledge user to understand their needs in more detail, in order to develop contextually appropriate interventions that meets the dynamic needs of modern society. Designing tools to facilitate healthy behavior change that is specific to individuals’ context, as well as tools and techniques to alleviate HCP workload to facilitate long-term buy-in of such programming could benefit from additional research. 

## Figures and Tables

**Table 1:** Demographic characteristics of participants.

Demographic Variables	CON	PPLP			
	Mean (SD)	Mean (SD)	*t*	*df*	*p*
Age (years)	61.3 (9.4)	57.7 (11.8)	1.14	45	0.257
Diabetes diagnosis (years)	8.4 (7.1)	5.8 (5.7)	1.41	42	0.166
	***N*** **(%)**	***N*** **(%)**	***X*^2^**	***df***	***p*** **Value**
Gender			2.481	1	0.115
Male	15 (60)	9 (37.5)			
Female	10 (40)	15 (62.5)			
Ethnicity			0.327	1	0.568
White	23 (92)	20 (87)			
Other	2 (8)	3 (13)			
Education			0.10	1	0.921
High school or less	8 (32)	8 (33)			
More than high school	17 (68)	16 (67)			
Employment Status			0.10	1	0.921
Working	12 (48)	10 (42)			
Other	13 (52)	14 (58)			
Household annual income			0.016	1	0.900
<$59,999	10 (42)	10 (43.5)			
>$60,000	14 (58)	13 (56.5)			
Annual income					
<Meet needs	7 (28)	6 (25)	0.9	-	-
>Meet needs	18 (72)	18 (75)		-	-

Baseline characteristics were compared between groups (CON vs. PPLP) using t-test for continuous variables and *X*^2^ test for categorical variables.

**Table 2:** Baseline metabolic characteristics.

Anthropometric Characteristics		CON	PPLP			
		Mean (SD)	Mean (SD)	*t*	*df*	*p*
Weight (kg)						
	Male	105.7 (16.4)	118.3 (46.7)	−0.96	22	0.345
	Female	90.1 (22.3)	92.7 (25.2)	−0.267	22	0.792
BMI (kg/m^2^)						
	Male	33 (4)	37(11)		22	0.219
	Female	34 (5)	35 (8)	−0.502	22	0.621
Waist circumference (cm)						
	Male	114.3 (11.7)	121.3 (28)	−0.861	22	0.398
	Female	104.5 (17)	108.5 (13)	−0.659	23	0.516
SBP (mmHg)						
	Male	141 (12)	126 (13)	2.7	22	0.013
	Female	142 (20)	138 (21)	0.417	23	0.680
DBP (mmHg)						
	Male	82 (10)	78 (10)	0.780	22	0.444
	Female	79 (9)	79 (8)	0.033	22	0.974
Fat mass (%)						
	Male	31.0 (4.8)	33.5 (11.7)	−0.710	21	0.486
	Female	39.4 (5.2)	40.7 (8.3)	−0.416	20	0.682
Fat mass (kg)						
	Male	32.8 (9.4)	36.9 (19.2)	−0.710	21	0.486
	Female	34.9 (10.4)	36.7 (15.9)	−0.305	20	0.763
Fat free mass (kg)						
	Male	70.9 (8.5)	65.9 (9.2)	1.3	21	0.206
	Female	52.9 (13.4)	49.9 (7.4)	0.673	20	0.509
**Metabolic Characteristics**		**Mean (SD)**	**Mean (SD)**	**t**	***df***	***p***
HbA1c (%)						
	Male	6.9 (0.9)	7.5 (1.1)	−1.40	22	0.175
	Female	7.3 (1.2)	6.7 (0.6)	1.38	22	0.179
Triglycerides (mmol/L)						
	Male	2.2 (1.2)	3.4 (1.6)	−1.84	22	0.078
	Female	3.0 (1.1)	2.8 (1.1)	0.435	23	0.667
Total Cholesterol (mmol/L)						
	Male	3.6 (0.7)	3.9 (1.2)	−0.759	22	0.456
	Female	4.4 (0.9)	4.3 (1.3)	0.163	23	0.872
LDL-C (mmol/L)						
	Male	1.7 (0.7)	1.6 (1.5)	0.172	13	0.866
	Female	2.0 (0.5)	1.7 (0.7)	0.865	18	0.399
HDL-C (mmol/L)						
	Male	1.0 (0.2)	0.9 (0.1)	0.997	22	0.330
	Female	1.3 (0.4)	1.1 (0.2)	0.965	23	0.345

Baseline characteristics were compared between groups (CON vs. PPLP) using independent *t* test.

**Table 3:** Anthropometric and metabolic changes from baseline to 3- and 6-month follow ups with differences within and between groups over time.

Anthropometric Characteristics	Control Group		PPLP Group		Mean Differences (95% CI) between Group × Time			
	Mean Change (95%) from Baseline		Mean Change (95%) from Baseline					
	3-Month	6-Month	3-Month	6-Month	3-Month	*p* Value	6-Month	*p* Value
Weight (kg)	−0.660 (−1.81 to 0.48)	−0.55 (−2.23 to 1.13)	−1.56 (−2.87 to −0.24) *	−2.43 (−3.63 to −1.23) **	−0.89 (−2.58 to 0.79)	0.290	−1.88 (−3.92 to 0.154)	0.069
BMI (kg/m^2^)	−0.20 (−0.59 to 0.19)	−0.24 (−0.81 to 0.32)	−0.70 (−1.2 to −0.14) *	−0.90 (−1.41 to −0.39) *	−0.50 (−1.14 to 0.13)	0.118	−0.65 (−1.40 to 0.09)	0.084
WC (cm)	−1.05 (−3.42 to 1.32)	4.19 (1.9 to 6.4) **	3.39 (0.64 to 6.14) *	2.10 (−1.89 to 6.09)	4.44 (0.93 to 7.96)	0.015 *	−2.09 (−6.31 to 2.13)	0.322
SBP (mmHg)	−11.9 (−19.1 to −4.8) *	−9.5 (−16.5 to −2.4) *	−8.72 (−15.89 to 1.56) *	−5.80 (−13.75 to 2.15)	3.25 (−6.71 to 13.23)	0.512	3.70 (−6.49 to 13.90)	0.465
DBP (mmHg)	−5.0 (−9.2 to −0.8) *	−2.2 (−7.1 to 2.6)	−2.50 (−6.39 to 1.39)	−5.00 (−9.31 to −0.69) *	2.54 (−3.23 to 8.32)	0.378	−2.72 (−9.08 to 3.64)	0.390
Fat mass (%)	−0.24 (−1.36 to 0.87)	0.28 (−0.42 to 1.00)	0.51 (−1.14 to 2.17)	1.37 (−0.70 to 3.45)	0.75 (−1.09 to 2.61)	0.412	1.08 (−1.08 to 3.25)	0.308
Fat mass (kg)	−0.47 (−1.49 to 0.54)	0.14 (−0.92 to 1.20)	−0.30 (−1.76 to 1.16)	0.30 (−1.38 to 1.98)	0.17 (−1.48 to 1.83)	0.216	0.15 (−1.69 to 2.00)	0.863
Fat free mass (kg)	−0.13 (−1.49 to 1.21)	−0.45 (−1.63 to 0.71)	−1.59 (−2.81 to −0.37) *	−2.92 (−4.83 to −1.01) *	−1.45 (−3.32 to 0.41)	0.122	−2.46 (−4.54 to −0.38)	0.021 *
**Metabolic Characteristics**								
HbA1c (%)	−0.04 (−0.27 to 0.17)	−0.09 (−0.41 to 0.22)	−0.15 (−0.38 to 0.08)	0.06 (−0.26 to 0.38)	−0.10 (−0.41 to 0.21)	0.510	0.15 (−0.28 to 0.59)	0.474
Triglycerides (mmol/L)	−0.05 (−0.43 to 0.33)	−0.17 (−0.58 to 0.23)	−0.17 (−0.73 to 0.37)	−0.44 (−1.15 to 0.27)	−0.12 (−0.76 to 0.50)	0.686	−0.26 (−1.04 to 0.50)	0.484
Total Cholesterol (mmol/L)	0.02 (−0.30 to 0.34)	−0.10 (−0.42 to 0.27)	0.18 (−0.46 to 0.84)	−0.40 (−1.01 to 0.21)	0.16 (−0.49 to 0.83)	0.616	−0.29 (−0.92 to 0.33)	0.346
LDL-C (mmol/L)	−0.05 (−0.35 to 0.24)	0.07 (−0.11 to 0.26)	−0.01 (−0.48 to 0.46)	−0.16 (−0.57 to 0.24)	0.04 (−0.46 to 0.54)	0.862	−0.24 (−0.60 to 0.12)	0.183
HDL-C (mmol/L)	0.02 (−0.04 to 0.09)	0.04 (−0.04 to 0.12)	0.05 (−0.13 to 0.24)	−0.02 (−0.07 to 0.01)	0.0. (−0.14 to 0.21)	0.714	−0.06 (−0.16 to 0.02)	0.165

Mean differences within and between groups are presented as mean (95% confidence interval). A negative value indicates a decrease from baseline to 3 months and baseline to 6 months. Paired t-test was used to calculate the mean change within groups. Between groups, PPLP was subtracted from CON; thus, a negative value indicates a bigger change from baseline for the PPLP group. Independent t-test was used to calculate the mean difference between groups (CON vs. PPLP) at 3- and 6-month time points. * Significant difference at *p* < 0.05 and ** significant difference at *p* < 0.001.

**Table 4:** Diet quality, self-efficacy, and quality of life change from baseline to 3- and 6-month follow ups, with differences in within and between groups over time.

Diet Quality and Adherence Variables	Control Group			PPLP Group			Mean Differences (95% CI) between Group × Time			
	Mean (SD)	Mean Change (95%) from Baseline		Mean (SD)	Mean Change (95%) from Baseline					
	Baseline	3-Month	6-Month	Baseline	3-Month	6-Month	3-Month	*p* Value	6-Month	*p* Value
PDAQ (Score 7 max)	3.5 (0.9)	0.3 (−0.1 to 0.7)	0.3 (−0.03 to 0.8)	3.7 (0.9)	0.4 (0.1 to 0.8) *	0.5 (0.06 to 0.9) *	−0.3 (−0.9 to 0.2)	0.271	−0.1 (−0.9 to 0.5)	0.605
DSES (Score 8 max)	7.0 (1.8)	0.04 (−0.7 to 0.8)	0.68 (−0.3 to 1.6)	6.2 (1.7)	0.7 (−0.03 to 1.5)	0.3 (−0.5 to 1.2)	−0.03 (−1.09 to 1.01)	0.940	0.5 (−0.4 to 1.5)	0.277
EQ-VAS (Score 100 max)	68 (18.7)	7.3 (−0.1 to 14.8) *	7.5 (3.1 to 11.8) *	67.8 (15.2)	5.3 (0.4 to 10.2) *	3.9 (−2.5 to 10.4)	3.4 (−5.0 to 11.8)	0.416	5.3 (−5.4 to 16.2)	0.321

Mean differences within and between groups are presented in mean (95% confidence interval). Within group, a negative value indicates a decrease from baseline to 3 months and baseline to 6 months. Paired t-test was used to calculate the mean change within groups. Between groups, PPLP was subtracted from CON; thus, a negative value indicates a bigger change from baseline for the PPLP group. Independent t-test was used to calculate the mean difference between groups (CON vs. PPLP) at 3- and 6-month time points. * Significant difference at *p* < 0.05.

**Table 5:** Qualitative analysis, representative quotes for key facilitators/barriers.

Facilitators and Barriers	
Themes	Example Quotes
1. Knowledge acquired	
Healthy eating	*I spend a lot more time thinking about my menu for the week, versus just grabbing whatever out of the fridge. So when I go shopping, I do it with more intent, when I’m doing my cooking for the week, I do it with more intent [PPLP-5B].*
Label reading	*I had no idea, uh, one thing I really benefited from was reading labels. I, um, I had no idea how much salt was in processed foods, uh, and sugars, and that was a real benefit to me [PPLP-1B].*
Physical Activity	*I think awareness of activity level … is very beneficial because it, it did make you realize that you have days where you might walk fifteen hundred steps, which is barely enough to keep you alive [PPLP-7B].*
2. Motivation	
Health	*My biggest thing was to try and keep control on the diabetes with food and that because I don’t want to go any further with medications. I’m scared of going on insulin [PPLP-1A].*
	*I’m getting older and I was afraid I was gonna get my legs cut off and I was gonna go blind and therefore I needed to find something to, to make sure I’m going down the right path [PPLP-7B].*
Family	*I just wanna be able to keep control so that I can stay healthy and that, because you know, like uh, I’m waiting…my son’s getting married, hopefully within the next ten years I’ll be a grandfather, I’d like to be pretty healthy to play with the grandkids and stuff like that [PPLP-1A].*
Group support	*I think what worked well for me was coming every week and being, like I said, accountable, and then hearing other people doing really well, and then feeling guilty that I didn’t do really well and then the next week I felt…well I gotta…they’re all doing it, I gotta step up and be accountable [PPLP-2B].*
3. Barriers	
Mental	*I’ve been able to go and buy the food and that, but it’s uh, for me it’s just a lot of laziness at times when I’ll be driving home and thinking it’s easier to stop and buy A&W and pick up a burger than trying to go through the hassle of making something at home and that. So, I still eat out way too much than I should and that [PPLP-4A].* *I find that you know, to cook it right before I have to eat it, I hahaha…I don’t do that you know. Like, I just want it ready so I can just take it out and you know, don’t spend the hour cooking … we do get lazy and guilt-ridden and uh you know whiny and that sort of thing [PPLP-3A].* *I’ve been really depressed and, so it’s been hard to think of…to take care of myself [PPLP-5A].*
Mechanical/metabolic	*Having MS [multiple sclerosis] and being immobile, I, my health really depends on what I eat, like I can’t control my weight or anything with movement, so I can’t exercise or burn calories that way [PPLP-6A].*
Monetary	*Sometimes you don’t get home from work and you haven’t had the time to plan so I, like I often felt like I need to take time to plan out the meals, to get the groceries, to…but if you don’t have the time to get ahead of yourself like that, then the time is working against you [PPLP-2B].* *Financially, like I haven’t really had much control over the food that’s been coming into my home [PPLP-3B].* *Buying fresh produce and, and uh…and fresh, fresh ingredients is much more expensive [PPLP-5B].*

**Table 6:** Qualitative analysis, representative quotes for key program evaluation experiences.

Experience Evaluation	
Themes	Example Quotes
1. Satisfaction	*Practical and easy to understand and when you take a look at other, uh, books and programs, it becomes very complicated, but this one was, was laid out, I think in, um easy to understand and very practical terms [PPLP2A].* *I find the best benefit is that…giving you the alternatives, like… saying don’t eat this, don’t do this…here is something you can…have instead…[PPLP5B]* *I just enjoyed all the sessions. I learned a lot, you know like I went for…to a dietitian, and it was one day, you know like what can you…learn all this stuff in one day so this is spread over time and you know with the group session, that’s what I really like about it [PPLP4A].* *This made me very conscious of my overall lifestyle* *[PPLP-2A].*
2. Limitations	*Honestly the paperwork. Like the recording of everything, I didn’t do it. I, I do enough paperwork at work that when I, you know, spend…to come home and spend another twenty minutes, half an hour on that when I’m already doing lots of other work. This was a deterrent. If this was an app, it would be beneficial [PPLP-5B].*
3. Improvements Support group	*I think it would be nice that a core group has been established. If you’re feeling like it’s just, uh, overwhelming, or you’re just, you’re not doing as well as you anticipated, to be able to be a part of an evening group that does something, uh nutrition or whatever the program might be. And so that gives you that little bit of an extra boost [PPLP-2A].*
Hands on activities	*Active cooking classes for next [PPLP-6B].*
Active PA	*Set up an exercise program for you that would be sort of matched to um your goals, yeah, and your abilities [PPLP-2B].*

**Table 7:** Qualitative analysis, representative quotes of health care providers (HCPs).

**Examples of HCPs Views of the Intervention**
*“I would rank it high, um I, I think…I was personally very pleased. I would rank it five. Like very high. Uh, I thought everything…I thought it was a…in my opinion, it was a quality um program” [PPLP-HCP1].* *“I’d say probably about a four [PPLP-HCP2].”*
*“When we’re talking to a lot of our patients regarding diabetes, we talk a lot about um either exchanging…like the carb choices or um grams of carbohydrate and um I know the Pure Prairie Eating Plan cookbook is based on Canada’s Food Guide. That to me makes better sense in a lot of ways and it’s easier, it’s just wrapping our heads around thinking a little bit differently” [PPLP-HCP1].*
*“I think, because there was uh, like [RD] and I, there was two of us kind of working on implementing it, that was helpful cause we um tend to be on the same page with a lot of things. And she could take some tasks and I could take some tasks and we could kind of get it done together“ [PPLP-HCP2].* *“Uh well a lot of things went well, um I think just, [RD] and I were able to work together very well um with your help and [Research member’s] help…. It was a lot of work, but I think it really worked out well. That we really had, I think there was a lot of agreement, with how um things should look, how the presentation should, how the slides should look, how the presentation, how the slides um should be presented. And I think it was nice to have the support from the team, uh the U of A, um our, our girls out front received everybody here” [PPLP-HCP1].*
**Examples of HCPs Challenges of the Intervention**
*“The calls were a lot of work haha. A lot more work than we anticipated… For the recruitment and for the follow-ups, yeah… it was a lot of administrative time on our part, and it took away from the rest of our patient time ”[PPLP-HCP2].*
*“Definitely trying to schedule the boardrooms, cause we’re in competition with all of our regular classes plus our mental health classes plus there’s board meetings, there’s um, Alberta Health Services uses our board rooms for some of their programming too, so there’s…it’s a challenge… to, to find it, and especially cause it…when you need the same board room consistently at the same time for several weeks in a row…that was the big challenge. We could, you know, here and there you can always find an empty boardroom, but um the multiple weeks in a row was very challenging to get the space” [PPLP-HCP2].*
